# Supplementary material for: Conventional video-based system for measuring the subtask speed of the Timed Up and Go Test in older adults: Validity and reliability study
Source: PLoS One. 2023 Jun 2;18(6):e0286574. doi: 10.1371/journal.pone.0286574 (PMC10237454; doi:10.1371/journal.pone.0286574)
Supplement: S1 File — Means of movement speed in nine TUG subtasks in both the comfortable and fast speed conditions from the video-based system and motion analysis system for each participant. (PDF) [file pone.0286574.s001.pdf]

Subject ID	Testing conditions	Motion analysis sit-to-stand (m/s)	Video sit-to-stand (m/s)	Motion analysis meter 0-1 (m/s)	Video meter 0-1 (m/s)	Motion analysis meter 1-2 (m/s)	Video meter 1-2 (m/s)	Motion analysis meter 2-3 (m/s)	Video meter 2-3 (m/s)	Motion analysis turning (m/s)	Video turning (m/s)	Motion analysis meter 3-2 (m/s)	Video meter 3-2 (m/s)	Motion analysis meter 2-1 (m/s)	Video meter 2-1 (m/s)	Motion analysis meter 1-0 (m/s)	Video meter 1-0 (m/s)	Motion analysis stand-to-sit (m/s)	Video stand-to-sit (m/s)
S1	Comfortable speed	0.358	0.384	0.948	0.759	1.048	0.972	0.927	0.872	0.488	0.345	0.998	0.774	1.091	0.880	0.848	0.583	0.287	0.280
S2	Comfortable speed	0.385	0.300	1.091	0.944	1.165	1.007	0.928	0.797	0.259	0.240	0.987	1.020	1.094	1.067	0.834	0.774	0.225	0.188
S3	Comfortable speed	0.253	0.198	1.073	1.007	1.165	1.173	0.983	0.988	0.418	0.360	1.049	0.856	1.153	0.961	0.888	0.683	0.168	0.117
S4	Comfortable speed	0.300	0.359	0.926	0.765	1.002	0.805	0.679	0.541	0.224	0.183	0.809	0.785	0.971	0.949	0.748	0.685	0.189	0.184
S5	Comfortable speed	0.280	0.340	0.997	0.797	1.090	0.883	0.859	0.668	0.433	0.348	1.013	0.958	1.022	1.012	0.604	0.593	0.160	0.149
S6	Comfortable speed	0.509	0.463	1.320	0.984	1.320	1.150	1.022	0.853	0.588	0.531	1.257	1.137	1.238	1.096	0.991	0.759	0.267	0.250
S7	Comfortable speed	0.396	0.358	1.208	0.920	1.144	1.061	0.945	0.745	0.590	0.479	1.059	0.978	1.151	0.988	0.852	0.618	0.332	0.263
S8	Comfortable speed	0.396	0.374	1.337	1.071	1.357	1.183	0.959	0.768	0.481	0.467	1.211	1.159	1.320	1.180	0.847	0.706	0.323	0.181
S9	Comfortable speed	0.357	0.281	1.254	1.009	1.323	1.113	0.934	0.724	0.393	0.222	1.093	0.909	1.257	1.114	1.085	0.823	0.200	0.169
S10	Comfortable speed	0.379	0.388	1.206	0.937	1.299	1.130	1.136	0.979	0.660	0.458	1.201	1.017	1.276	1.094	1.020	0.737	0.292	0.302
S11	Comfortable speed	0.365	0.405	1.207	1.027	1.273	1.189	0.959	0.996	0.393	0.388	1.089	0.914	1.234	1.067	0.870	0.727	0.249	0.208
S12	Comfortable speed	0.406	0.476	1.056	0.923	1.166	1.087	1.033	1.002	0.375	0.412	1.023	0.819	1.104	0.872	0.902	0.652	0.340	0.307
S13	Comfortable speed	0.312	0.356	1.231	1.085	1.304	1.254	1.062	1.111	0.466	0.512	1.162	0.973	1.246	1.097	0.920	0.762	0.264	0.277
S14	Comfortable speed	0.278	0.357	1.122	1.022	1.270	1.073	0.997	0.886	0.416	0.421	1.211	1.203	1.233	1.289	0.973	0.789	0.201	0.205
S15	Comfortable speed	0.458	0.409	1.116	0.959	1.240	1.028	1.012	0.842	0.407	0.410	1.061	1.058	1.158	1.163	0.824	0.617	0.406	0.272
S16	Comfortable speed	0.300	0.276	1.172	1.026	1.253	1.196	1.025	1.076	0.539	0.513	1.173	0.986	1.230	1.023	0.917	0.725	0.337	0.245
S17	Comfortable speed	0.229	0.202	0.782	0.650	0.803	0.637	0.774	0.641	0.345	0.323	0.810	0.791	0.846	0.794	0.599	0.574	0.227	0.143
S18	Comfortable speed	0.367	0.240	1.217	1.038	1.294	1.222	1.018	1.022	0.383	0.376	1.093	0.937	1.164	0.952	0.879	0.657	0.189	0.132
S19	Comfortable speed	0.397	0.322	1.091	0.995	1.251	1.041	0.999	0.941	0.409	0.376	1.114	1.178	1.258	1.217	0.959	0.794	0.240	0.162
S20	Comfortable speed	0.347	0.343	1.186	1.099	1.393	1.286	1.079	1.213	0.344	0.342	1.144	1.033	1.376	1.179	0.992	0.831	0.383	0.284
Mean		0.354	0.342	1.127	0.951	1.208	1.075	0.967	0.883	0.430	0.385	1.078	0.974	1.171	1.050	0.877	0.704	0.264	0.216
SD		0.069	0.075	0.138	0.121	0.141	0.159	0.104	0.171	0.107	0.096	0.120	0.131	0.125	0.126	0.123	0.081	0.072	0.061
Max		0.509	0.476	1.337	1.099	1.393	1.286	1.136	1.213	0.660	0.531	1.257	1.203	1.376	1.289	1.085	0.831	0.406	0.307
Min		0.229	0.198	0.782	0.650	0.803	0.637	0.679	0.541	0.224	0.183	0.809	0.774	0.846	0.794	0.599	0.574	0.160	0.117
S1	Fast speed	0.469	0.340	1.087	0.871	1.228	1.163	0.974	0.926	0.417	0.330	1.083	0.894	1.281	1.039	0.919	0.615	0.319	0.399
S2	Fast speed	0.293	0.296	1.260	1.019	1.339	1.119	1.031	0.882	0.280	0.286	1.118	1.103	1.360	1.376	1.235	0.951	0.342	0.248
S3	Fast speed	0.297	0.276	1.135	0.973	1.233	1.194	1.058	0.974	0.392	0.330	1.156	0.847	1.261	0.990	0.982	0.713	0.218	0.151
S4	Fast speed	0.392	0.414	1.155	0.930	1.237	0.973	0.828	0.683	0.338	0.292	1.023	1.022	1.203	1.171	0.877	0.779	0.223	0.175
S5	Fast speed	0.387	0.415	1.123	0.915	1.185	0.956	0.928	0.694	0.403	0.370	1.114	1.058	1.179	1.164	0.722	0.858	0.177	0.192
S6	Fast speed	0.514	0.473	1.573	1.154	1.641	1.399	1.134	0.930	0.575	0.553	1.434	1.414	1.658	1.489	1.422	1.045	0.328	0.236
S7	Fast speed	0.466	0.404	1.497	1.075	1.479	1.296	1.042	0.784	0.556	0.511	1.260	1.172	1.404	1.235	1.309	0.918	0.511	0.320
S8	Fast speed	0.445	0.388	1.468	1.188	1.451	1.227	1.059	0.811	0.437	0.297	1.277	1.099	1.401	1.209	0.865	0.747	0.360	0.274
S9	Fast speed	0.297	0.313	1.337	1.098	1.447	1.193	1.091	0.873	0.491	0.319	1.188	0.999	1.358	1.198	1.123	0.892	0.258	0.235
S10	Fast speed	0.504	0.442	1.711	1.338	1.799	1.556	1.646	1.381	0.861	0.748	1.791	1.521	1.862	1.591	1.439	1.111	0.264	0.245
S11	Fast speed	0.442	0.553	1.483	1.215	1.638	1.490	1.070	1.177	0.360	0.377	1.410	1.181	1.625	1.412	1.235	1.015	0.315	0.250
S12	Fast speed	0.440	0.390	1.447	1.185	1.588	1.485	1.176	1.167	0.448	0.320	1.210	1.048	1.417	1.137	1.231	0.823	0.398	0.386
S13	Fast speed	0.477	0.502	1.589	1.386	1.726	1.650	1.291	1.355	0.566	0.600	1.503	1.323	1.639	1.397	1.008	0.810	0.372	0.385
S14	Fast speed	0.357	0.318	1.408	1.206	1.442	1.287	1.313	1.067	0.392	0.390	1.278	1.366	1.427	1.387	1.168	0.875	0.233	0.184
S15	Fast speed	0.517	0.585	1.415	1.216	1.579	1.280	1.272	1.105	0.485	0.500	1.332	1.383	1.538	1.502	1.206	0.847	0.441	0.386
S16	Fast speed	0.335	0.325	1.371	1.221	1.435	1.390	1.134	1.215	0.590	0.561	1.328	1.239	1.454	1.177	1.109	0.925	0.294	0.294
S17	Fast speed	0.285	0.406	1.233	1.057	1.388	1.096	1.101	0.910	0.421	0.392	1.154	1.127	1.345	1.246	0.989	0.793	0.366	0.324
S18	Fast speed	0.350	0.303	1.429	1.181	1.553	1.544	1.059	1.162	0.340	0.350	1.191	1.035	1.396	1.138	1.073	0.808	0.227	0.112
S19	Fast speed	0.432	0.374	1.445	1.243	1.595	1.435	1.300	1.165	0.339	0.358	1.326	1.381	1.630	1.598	1.230	1.114	0.270	0.286
S20	Fast speed	0.397	0.259	1.213	1.121	1.369	1.335	1.047	1.127	0.322	0.433	1.122	1.003	1.333	1.149	0.982	0.873	0.351	0.198
Mean		0.405	0.389	1.369	1.130	1.467	1.303	1.128	1.019	0.451	0.416	1.265	1.161	1.438	1.280	1.106	0.876	0.313	0.264
SD		0.077	0.090	0.172	0.137	0.173	0.192	0.175	0.203	0.132	0.124	0.176	0.186	0.173	0.177	0.189	0.128	0.083	0.084
Max		0.517	0.585	1.711	1.386	1.799	1.650	1.646	1.381	0.861	0.748	1.791	1.521	1.862	1.598	1.439	1.114	0.511	0.399
Min		0.285	0.259	1.087	0.871	1.185	0.956	0.828	0.683	0.280	0.286	1.023	0.847	1.179	0.990	0.722	0.615	0.177	0.112
