# Supplementary material for: Conventional video-based system for measuring the subtask speed of the Timed Up and Go Test in older adults: Validity and reliability study
Source: PLoS One. 2023 Jun 2;18(6):e0286574. doi: 10.1371/journal.pone.0286574 (PMC10237454; doi:10.1371/journal.pone.0286574)
Supplement: S2 File — Means of movement speed in nine TUG subtasks in both the comfortable and fast speed conditions from two sessions using the video-based system for each participant. (PDF) [file pone.0286574.s002.pdf]

Subject ID	Testing conditions	Session 1 sit-to-stand (m/s)	Session 2 sit-to-stand (m/s)	Session 1 meter 0-1 (m/s)	Session 2 meter 0-1 (m/s)	Session 1 meter 1-2 (m/s)	Session 2 meter 1-2 (m/s)	Session 1 meter 2-3 (m/s)	Session 2 meter 2-3 (m/s)	Session 1 turning (m/s)	Session 2 turning (m/s)	Session 1meter 3-2 (m/s)	Session 2 meter 3-2 (m/s)	Session 1meter 2-1 (m/s)	Session 2 meter 2-1 (m/s)	Session 1meter 1-0 (m/s)	Session 2 meter 1-0 (m/s)	Session 1 stand-to-sit (m/s)	Session 2 stand-to-sit (m/s)
S1	Comfortable speed	0.300	0.280	1.180	1.168	1.284	1.293	1.056	1.004	0.414	0.337	0.971	0.996	1.137	1.058	0.883	0.877	0.311	0.302
S2	Comfortable speed	0.199	0.270	1.055	1.105	1.181	1.214	1.060	1.044	0.443	0.438	0.871	0.879	0.945	0.993	0.773	0.837	0.321	0.324
S3	Comfortable speed	0.209	0.189	1.239	1.234	1.333	1.361	1.189	1.176	0.549	0.507	1.038	1.079	1.167	1.193	0.925	0.923	0.295	0.277
S4	Comfortable speed	0.197	0.255	1.126	1.160	1.146	1.147	0.942	0.951	0.454	0.453	1.279	1.267	1.379	1.349	1.024	1.046	0.287	0.181
S5	Comfortable speed	0.354	0.346	1.112	1.153	1.082	1.082	0.889	0.877	0.436	0.548	1.146	1.200	1.238	1.256	0.904	0.893	0.532	0.442
S6	Comfortable speed	0.290	0.340	1.178	1.264	1.293	1.372	1.143	1.160	0.550	0.538	1.052	1.101	1.098	1.125	0.910	0.923	0.344	0.391
S7	Comfortable speed	0.181	0.179	0.702	0.786	0.681	0.759	0.684	0.711	0.348	0.361	0.838	0.896	0.862	0.932	0.687	0.796	0.284	0.208
S8	Comfortable speed	0.271	0.220	1.195	1.194	1.313	1.345	1.079	1.110	0.422	0.395	1.006	0.977	1.028	0.989	0.780	0.750	0.218	0.164
S9	Comfortable speed	0.239	0.262	1.102	1.327	1.111	1.330	0.981	1.143	0.510	0.441	1.225	1.380	1.341	1.518	0.999	1.254	0.430	0.229
S10	Comfortable speed	0.283	0.267	1.283	1.227	1.395	1.354	1.277	1.232	0.520	0.524	1.097	1.052	1.272	1.190	0.995	0.946	0.433	0.350
Mean		0.252	0.261	1.117	1.162	1.182	1.226	1.030	1.041	0.465	0.454	1.052	1.083	1.147	1.160	0.888	0.925	0.346	0.287
SD		0.056	0.055	0.161	0.146	0.204	0.192	0.168	0.160	0.066	0.074	0.141	0.161	0.168	0.181	0.110	0.142	0.093	0.092
Max		0.354	0.346	1.283	1.327	1.395	1.372	1.277	1.232	0.550	0.548	1.279	1.380	1.379	1.518	1.024	1.254	0.532	0.442
Min		0.181	0.179	0.702	0.786	0.681	0.759	0.684	0.711	0.348	0.337	0.838	0.879	0.862	0.932	0.687	0.750	0.218	0.164
S1	Fast speed	0.335	0.263	1.466	1.448	1.607	1.601	1.253	1.234	0.403	0.363	1.251	1.183	1.504	1.426	1.214	1.217	0.287	0.395
S2	Fast speed	0.254	0.252	1.451	1.466	1.592	1.583	1.240	1.260	0.352	0.486	1.116	1.168	1.208	1.268	1.015	1.121	0.305	0.374
S3	Fast speed	0.305	0.286	1.604	1.496	1.766	1.731	1.449	1.418	0.636	0.575	1.406	1.333	1.498	1.489	1.069	1.108	0.365	0.301
S4	Fast speed	0.281	0.284	1.377	1.338	1.354	1.339	1.124	1.105	0.516	0.548	1.445	1.454	1.504	1.577	1.127	1.114	0.306	0.260
S5	Fast speed	0.420	0.400	1.382	1.423	1.345	1.357	1.170	0.994	0.535	0.502	1.472	1.372	1.604	1.501	1.125	1.004	0.484	0.470
S6	Fast speed	0.303	0.330	1.391	1.447	1.498	1.587	1.278	1.342	0.517	0.528	1.276	1.277	1.269	1.302	1.125	1.037	0.587	0.522
S7	Fast speed	0.210	0.241	1.198	1.196	1.143	1.196	0.968	1.002	0.417	0.478	1.190	1.177	1.354	1.278	1.083	0.987	0.333	0.231
S8	Fast speed	0.263	0.342	1.373	1.427	1.651	1.606	1.233	1.243	0.376	0.390	1.105	1.108	1.222	1.288	1.004	1.080	0.197	0.261
S9	Fast speed	0.300	0.241	1.441	1.515	1.506	1.533	1.252	1.260	0.522	0.499	1.457	1.560	1.695	1.721	1.422	1.224	0.286	0.332
S10	Fast speed	0.266	0.261	1.278	1.363	1.439	1.531	1.206	1.377	0.468	0.552	1.068	1.177	1.232	1.345	1.059	1.091	0.354	0.355
Mean		0.294	0.290	1.396	1.412	1.490	1.506	1.217	1.223	0.474	0.492	1.279	1.281	1.409	1.419	1.124	1.098	0.350	0.350
SD		0.056	0.052	0.109	0.093	0.179	0.160	0.122	0.147	0.087	0.069	0.157	0.146	0.175	0.151	0.121	0.079	0.111	0.094
Max		0.420	0.400	1.604	1.515	1.766	1.731	1.449	1.418	0.636	0.575	1.472	1.560	1.695	1.721	1.422	1.224	0.587	0.522
Min		0.210	0.241	1.198	1.196	1.143	1.196	0.968	0.994	0.352	0.363	1.068	1.108	1.208	1.268	1.004	0.987	0.197	0.231
